# The effect of encapsulated plant extract of hyssop (*Hyssopus officinalis L*.) in biopolymer nanoemulsions of *Lepidium perfoliatum* and *Orchis mascula* on controlling oxidative stability of soybean oil

**DOI:** 10.1002/fsn3.1415

**Published:** 2020-01-13

**Authors:** Negin Rezaei Savadkouhi, Peiman Ariaii, Mehdi Charmchian Langerodi

**Affiliations:** ^1^ Department of Food Science and Technology Sari Branch Islamic Azad University Sari Iran; ^2^ Department of Food Science and Technology Ayatollah Amoli Branch Islamic Azad University Amol Iran; ^3^ Department of Agricultural Extension and Education Sari Branch Islamic Azad University Sari Iran

**Keywords:** encapsulation, hyssop extract, oxidation, soybean oil

## Abstract

The aim of this study was to investigate the effect of encapsulation method on antioxidant properties of Hyssop (*Hyssopus Officinalis* L.) extract. The extracts of the Hyssop were obtained by ultrasound assisted techniques, and the amount of phenolic compounds was 117.43 ± 9.22 (mg of gallic acid per 100 g of extract). The antioxidant activity of extracts in concentrations of 100, 200, 300, and 400 ppm was measured using DPPH free radical scavenging method and compared with 100 ppm of TBHQ synthetic antioxidants. The results showed that by increasing in concentration of the extract, the antioxidant activity of the extract increased. The *Lepidium perfoliatum* seed gum and *Orchis mascula* were chosen as coating material. Encapsulation was performed by emulsion production method. The antioxidant effects of nanocapsules in oil during 40 days of storage at 60°C were measured, which increased the oxidation of oil over time. The lowest amount of oil oxidation during storage compared to control samples was observed in samples containing nano encapsulated Hyssop extract due to reducing in release rate of the extract during storage and more protection of the extract. The results of this study suggest that encapsulation is an effective way to increase the antioxidant activity of the extract and could be increased the shelf life of edible oils with natural antioxidants.

## Graphical abstract

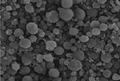

## 1 INTRODUCTION

Oxidation of oils and fats is one of the most important factors in the corruption of these compounds when exposed to high temperatures and also during storage, which, due to autoxidation of fats, causes a rancid odor, undesirable flavor, and inappropriate color. It reduces the nutritional value and safety of the product. The production of free radicals caused to damages cell wall membrane and leading to heart disease and cancer. One of the ways to prevent lipid oxidation or to protect against harm caused by free radicals is to use antioxidants (Wanasundara & Shahidi, 1998).

Along with the concern about the harmful effects of lipid oxidation on human health and food degradation, the need to use antioxidants is essential to prevent the effects of free radicals on the body and to prevent the degradation of oils. Commercial antioxidants, such as synthetic antioxidants and antioxidant supplements such as BHT and BHA, are widely available in the market and are used extensively in the world. The use of synthetic antioxidants may increase the risk of cancer and have negative effects on the consumer. Today, the use of plant derivatives has been developed as a natural antioxidant (Krishnaiah, Sarbatly, & Nithyanandam, 2010). Herbal extracts provide a unique range of health benefits. Secondary metabolites of plants, such as phenolic compounds derived from plant sources, play an important role in perseverating food as antioxidants (Sikwese & Duodu, 2007).

Hyssopus (*Hyssopus Officinalis* L.) from the family Lamiaceae is derived from the Greek word “azob” meaning “holy plant” and is one of the most important plant species. This plant was used from the old in order to make beverages flavored. In Iran, hyssopus plant is grown spontaneous in the provinces of East Azarbaijan, West Azarbaijan, Sistan and Baluchestan, and the regions around the Caspian Sea. The hyssopus extract have antifungal, antibacterial, antiviral (especially AIDS) properties and widely used in food industry and cosmetics. hyssopus has a warm and dry nature, and its leaves are reducing blood glucose, reinforcing the stomach, asthma treatment, emmenagogue, and very beneficial. Hyssopus succulent leaves are useful for the removal of the infection and healing of the wounds (Omid Beigi, 2006). Encapsulation is a technique which solid, liquid, or gas components are covered within a small capsule so that they can be released at controlled speed under specific conditions. There are many techniques and methods for the encapsulation of dietary bioactive compounds. However, three major targets, including the formation of a suitable wall around the selected material, preventing leakage and penetration of the encapsulated material into the capsule surface during storage, and the prevention of contact and reaction of the environmental factors with the core, are followed in all of these processes (Calvo & Santagapita, 2017; Javadian, Shahoseini, & Ariaii, 2017).

The low stability of phenolic compounds during the process and storage of food against environmental factors such as oxygen, heat, chemical and biological agents, low solubility in water, and bitter taste has limited the use of these compounds in foods. These problems can potentially be improved with encapsulation (Nedovic, Kalusevic, Manojlovic, Levic, & Bugarski, 2011). According to the importance of medicinal plant extracts in preventing oil oxidation, the aim of this study was to compare the antioxidant activity of the encapsulated hyssopus extract in biopolymer nanoemulsion in preventing soybean oil oxidation and enhancing its stability.

## 2 MATERIALS AND METHODS

### 2.1 Materials

Hyssopus plant was collected from the farms of Mazandaran province in the autumn 2018 and identified by Professor Yousef Niknejad from Department of Botany, Ayatollah Amoli Branch, Islamic Azad University, Amol, Iran. After cleaning the leaves of the hyssopus, they dried in an oven under vacuum at 40°C until it reached constant weight and then turned into powder with the mill (Dahmoune, Nayak, Moussi, Remini, & Madani, 2015). *Orchis mascula* (Salab) and *Lepidium perfoliatum* (Ghodumeh) seed were purchased from grocery of Sari. The seed gum after purchase was cleaned in order to remove foreign matter such as thorns, stones, broken grains, and straw. Soybean oil without antioxidants was obtained from Rana Golestan oil factory. All the chemicals used in this study have analytical grade and were obtained from the Merck Co. of Germany.

### 2.2 Methods

#### 2.2.1 Plant extraction by ultrasound

Ten grams of crushed hyssopus samples was extracted with 100 milliliters of ethanol: water (80%) at 45°C and time (20 min) in ultrasonic bath at 20 KHz. Then, the solvents evaporated by under vacuum evaporator (Esmaeilzadeh Kenari, Mohsenzadeh, & Amiri, 2014).

#### 2.2.2 Total phenolic compound of the extract

In order to measure the phenolic compounds of extract, 0.1 ml of the extract solution (at a concentration 1 mg/ml) was mixed with 0.5 ml of folin ciocalteu (diluted with distilled water at a ratio of 1 to 10). After 1 to 8 min, 0.4 ml of sodium carbonate solution (7.5% w/v) was added to the mentioned test tube. The test tubes after shaking were putted in a water bath at 40°C, and after 30 min, their optical absorption was measured at 765 nm with a spectrophotometer. Finally, the data were expressed according to the standard gallic acid equation and based on mg of gallic acid per 100 g of extract (McDonald, Prenzler, Antolovich, & Robards, 2001).

#### 2.2.3 Measuring the antioxidant activity of the extract

0.3 ml of a hyssop extract with different concentrations was mixed with 2.7 ml of methanolic solution of DPPH (6 × 10^–5^) and stored for 60 min at room temperature in a dark place and absorbance at 517 nm were read. For the control sample, all the compounds and conditions mentioned were used without adding extracts. The following formula has been used to control the free radicals (Esmailzadeh Kenari et al., 2014). Synthetic antioxidant TBHQ was used as a positive control at 100 ppm.

(1) $$\begin{array}{c} \%\;\text{DPPH free radical scavenging}=\left(\text{Sample absorbance}-\text{DPPH}\right)\\ /\text{DPPH absorbance} \end{array}$$

The 300 ppm of extract was used for injection into oil and encapsulation.

#### 2.2.4 Extraction of gum

The gum of Lepidium perfoliatum seed were extracted according to Koocheki, Ghandi, Razavi, Mortazavi, and Vasiljevic (2009). Optimum extraction conditions include water to seed ratio 1:30, temperature 48°C, and pH = 8. During gum extraction process, the pH of deionized water was first adjusted by NaOH 0.1 molar or HCl solution and it was heated in the hot water bath until to reach the desired temperature, and then, the seeds were added to it. For complete absorption process, it was putting in the hot water bath and was stirred intermittently. Finally, the hydrocolloid extract was obtained by a laboratory extractor and dried in an oven at 70°C. Then, the mill and sieve (with mesh 1 mm) and the gum powder stored in closed containers in a dry place for the desired experiments.

#### 2.2.5 Preparation of emulsion

The composition of Lepidium perfoliatum seed gum and Orchis mascula (1:1) was used as a continuous phase of emulsion. The Lepidium perfoliatum seed gum was mixed with 1:1 Orchis mascula to get a 30% of total solid material in deionized water. The magnetic stirrer was used for 15 min at ambient temperature to better dissolve the compounds. The solution was stored in the refrigerator for 24 hr to complete the water absorption process. The hyssopus extract was added to the gum solution in 5:1 ratio (continuous aqueous phase). 20 grams of soybean oil without antioxidant (dispersed oil phase) was added to the solution. At this stage, 80% emulsifiers were used as surfactants.

The emulsion preparation method was performed according to Weiss et al. (2008). The two phases were separately heated to 35°C, and initial emulsion formed by a magnetic mixer. In order to reduce the particle size to the nanoscale, ultratorax homogenizer at 12,000*g* at 10°C was used for 5 min to homogenization. For more reduction of particle size, ultrasonic generator probe type with 6‐cycle propulsion, the time of each cycle was 30 s, and the rest time was 15 s between cycles was used (Carneiro, Tonon, Grosso, & Hubinger, 2013). For drying of nanocapsules, a freeze drying method was used at 0.017 mA pressure and −57°C for 48 hr (Chranioti, Nikoloudaki, & Tzia, 2015).

#### 2.2.6 Particle size measurement of capsules

The average diameter of the capsules (D3,2) was measured using the Shimadzu Particle Size Analyzer (SALD‐2101 Japan) based on laser light diffraction. Distilled water was used as a reference. All samples were measured in three replicates.

(2) $$D\left[3,2\right]=\frac{\Sigma nidi^{3}}{\Sigma nidi^{2}}$$

Where n_i_: the number of particles, d_i_: average particle diameter, D: Average volume diameter (average volume of equivalent).

#### 2.2.7 Encapsulation efficiency

The encapsulation efficiency of polyphenols was determined according to the method described by Robert et al. (2010). 200 milligrams of nanocapsules was added to 2 ml of ethanol and washed for 1 min, then treated with ultrasound (Chroma tech, Taiwan) for 20 min in two steps of 100 percent intensity and 20 kHz frequency. After this, centrifuging was carried out at 3,000*g* for 2 min. Alcohol can dissolve the extract on capsule surface without destroying it. The total phenolic compounds in the supernatant were determined using the folin ciocalteu method and absorbed at 740 nm was evaluated by spectrophotometer. The percentage of encapsulation efficiency is calculated from the following equation:

(3) $$\text{Encapsulation Efficiency}\;\left(\%\right)\;=\left(w_{1}-w_{2}\right)/w_{2}$$

Where w_1_ is the amount of extract on the upper liquid of nanocapsule, and w_2_ is of the amount of extract added to prepare the same amount of nanocapsule.

#### 2.2.8 Morphology of nanocapsules

To investigate the morphology and confirm their size on a nanoscale (smaller than 100 nm), scanning electron microscopy the Tescan, Mira3 FEG model was used. A small amount of sample was placed with silver adhesive on an aluminum stap and then placed on a thin layer of gold in a coating machine for 6 min to be conductive. Samples were transferred to a vacuum chamber. Radius of accelerated electrons with a voltage of 20 kV was radiated to the samples, and the image was obtained based on the electron beam returning from the samples.

#### 2.2.9 Oil tests

Pure extract and nanoencapsulated hyssopus extract at 300 ppm were added to soybean oil without antioxidant and stored for 40 days at 60°C. Chemical oil tests were carried out at 8 day intervals.

#### 2.2.10 Measurement of peroxide value and thiobarbituric acid value

To measure the peroxide value (PV), the AOCS method No (Cd 8–53) was used and PV expressed in milligrams of peroxide per 1,000 grams of oil (AOCS, 1990). The thiobarbituric acid value (TBA) was performed according to the AOCS method No (Cd 19–90). The absorbance of the samples was read at 530 nm by spectrophotometer and reported in milligrams of malon dialdehyde per kg of oil (AOCS, 1990).

### 2.3 Statistical analysis of data

Statistical analysis of the data obtained from different methods was carried out using two‐way ANOVA in a completely randomized design with Duncan test at confidence level 95%. SPSS software version 20 was used, and in order to reduce the error, all tests were performed in three replications.

## 3 RESULTS AND DISCUSSION

### 3.1 Total phenolic compounds and antioxidant activity of hyssopus extract

Hyssopus is one of the most important herbs used in the pharmaceutical industry. Despite the fact that the plant has a bitter taste, it is used in the food industry as a food flavor agent. The application of this plant in the medical industry is used for the antifungal, detomidine, and analgesic use of the stomach, which has not been mentioned in all scientific applications (Ebrahimzadeh, Nabavi, Nabavi, Bahramian, & Bekhradnia, 2010; Fathiazad & Hamedeyazdan, 2011; Kizil, Hasimi, Tolan, & KARATAS, H., 2010). The total phenol content of the extract was reported 117.43 ± 9.22 mg GA/100 g E. In a study by Vlase et al. (2014), the amount of phenolic compounds extracted in the hyssopus extract, which was obtained using the ethanol solvent and maceration method, was 77.72 mg/g, indicating that the plant due to have phenolic compounds shows antioxidant and medicinal properties. The reason for the higher amount of phenolic compounds of extracts obtained by ultrasound assisted technique is the cavitation effects of ultrasound waves. Also, mechanical destruction of the cell wall results in more soluble penetration in plant tissues, which helps to further penetrate the phenolic compounds of the extract in plant tissues (Hussain et al., 2012). Many researchers believe that ultrasound extraction due to less time consumes increase the extraction efficiency and the extract which achieved by this has higher phenolic compounds than extracts obtained by the solvent method (Khan, Abert‐Vian, Fabiano‐Tixier, Dangles, & Chemat, 2010; Teh & Birch, 2014). The amount of phenolic compounds of a plant depends on the type of plant, environment, climate, genetic factors, soil type, harvest season, drying and maintenance conditions and extraction methods, and the quantity and quality of the measured compounds. (Benedec, Vlase, Hanganu, & Oniga, 2012).

The chemical composition of the hyssopus extract has been investigated in some studies, and the presence of phenolic compounds such as Isopinocamphene, camphene, and myrcene has been proven in this plant (Mitić, V, & Đorđević, 2000). The ethanolic extract of the hyssopus plant contains compounds such as chlorogenic acid, isocoerythritin, routine, quercetin, rosemary acid, patholtin, and all of which are phenolic compounds and their antioxidant properties have been proven in previous studies (Vlase et al., 2014).

Antioxidant activity is one of the most important parameters for plant extracts. The acidification caused to the destruction of biological materials and thus causes diseases such as cancer, Alzheimer's, digestive diseases, diabetes, and Parkinson's disease in the body (Shaaban, El‐Ghorab, & Shibamoto, 2012). In other words, plant extracts are due to the presence of phenolic compounds have antioxidant activity and high capacity for donating hydrogen atoms to electrons and free electrons (Demirci, Koşar, Demirci, Dinç, & Başer, 2007). In Table 1, with increasing concentrations of hyssopus extract, the DPPH free radicals scavenging was increased and a statistically significant difference (*p* < .05) was observed. The antioxidant activity of 300 ppm concentration of hyssopus extract was not statistically significant with 100 ppm of synthetic antioxidant TBHQ.

**Table 1:** Antioxidant activity of hyssopus hydroalcoholic extract using DPPH free radical scavenging

	100	200	300	400	TBHQ
DPPH free radical scavenging (%)	26.71 ± 2.9^d^	48.16 ± 3.56^c^	79.04 ± 2.73^b^	93.27 ± 4.05^a^	78.87 ± 3.15^b^

Note

The nonsimilar letters indicate a significant statistical difference (*p* < .05) between the various samples.

Electron donation is one of the important mechanisms in which phenolic compounds by converting of free radicals to nonradical forms, end the reactions of the radical chain (Zou, Opara, & McKibbin, 2006). Antioxidant activity of phenolic compounds is mainly due to its oxidation and reduction characteristics. Phenolic compounds act as a reducing agent, a hydrogen donator, and an active oxygenating agent and shows antioxidant effects (Oke, Aslim, Ozturk, & Altundag, 2009). The amount of antioxidant activity of the extracts in food systems is related to their concentration (Berger, 2007). By increasing the concentration of the extract or the degree of hydroxylation of the phenolic compounds, the DPPH free radical scavenging activity is increased, which is defined as antioxidant activity. (Ferreres et al., 2007; Sanjes Moreno, Larrauri, & Saura‐ Calixto, 1999). Other researchers also considered the activity of scavenging free radicals in proportion to the concentration of extracts (Alothman, Bhat, & Karim, 2009; Arabshahi‐Delouee & Urooj, 2007; Liu & Yao, 2007; Sun, Zhang, Lu, Zhang, & Zhang, 2011).

According to the results of antioxidant activity measurements, the concentration of 300 ppm of hyssopus because of not having statistically significant differences with the synthetic antioxidant TBHQ was selected for emulsion production and injection into oil.

### 3.2 Particle size of Nanocapsules and encapsulation efficiency

The formation of nanoemulsion droplets results from the interaction of two phenomena including droplet breaking and the formation of droplets’ conjugation. The use of ultrasound leads to providing the shear force needed to break droplets (Li & Chiang, 2012). Phenolic compounds are unstable compounds that are sensitive to environmental factors. Encapsulation protects these unstable components from temperature, humidity, and pH during foods processes until they are released in the system. On the other hand, encapsulation prevents of intense and unwanted flavors that phenolic compounds produce in foods (Sanchez, Baeza, Galmarini, Zamora, & Chirife, 2011). The average size of nanocapsule particles was 56.713 ± 1.48 nm. The particle size is one of the most important qualities of emulsions. The average diameter of the particles in the stability of the emulsions is very important (McClements, 2015). Generally, polysaccharides in emulsion systems play the role of condensation of the aqueous phase of emulsion (Dickinson, 1992), thus by increasing in concentration and viscosity of the continuous phase prevent the growth and dispersion of oil particles (Yuan, Gao, Mao, & Zhao, 2008). As explained in the materials and methods section, in this study, for the purpose of reducing the size of the ultraturrax homogenizer and the probe generator, both of them were involved in reducing the particle size, resulting in the formation of small particles (Tadros, 2009).

The encapsulation efficiency of the hyssopus extract in the composite biopolymer was 88.36 ± 2.4. Wu et al. (2008), using the encapsulation method, indicated the efficiency of quercetin encapsulation by 94%. Due to the fact that salab is composed of glucomannan units and has charged groups, it creates hydrophilic parts. Ghodumeh also due to its favorable emulsification characteristics had high encapsulation efficiency (Kaushik & Roos, 2007; Szente & Szejtli, 2004). The emulsions consist of hydrophobic and hydrophilic parts that hydrophilic compounds are enclosed in the aquatic environment and hydrophilic compounds in phospholipid layers (Heurtault, Saulnier, Pech, Proust, & Benoit, 2003).

### 3.3 Morphology of nanocapsules

**Figure 1:**
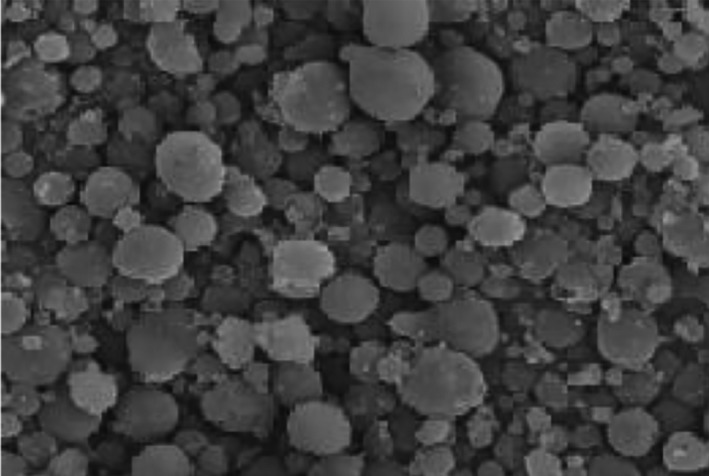
The scanning electronic microscopes of the hyssopus extract nanocapsules in the composite coating

The effectiveness of the encapsulation process depends on the creation of a spherical structure and confirmation of the trapping of the effective core compounds (López‐Córdoba, Deladino, Agudelo‐Mesa, & Martino, 2014). The image of the scanning microscope of encapsulated hyssopus extract in the composite coating is shown in Figure 1. Suitable network and uniform wall cover around the extract are very effective in maintaining the extract in the capsule, the rate of sedimentation, and release of extract from nanocapsules during the storage period. As shown in Figure 1, the particles are all spherical. Dent and fracture in the structure of nanocapsules were not observed. Various factors affect the surface properties of the nanocapsule wall, including the rapid drying of the nanoemulsion, the combination of coating, and the conditions of the nanoemulsion production (Lee & Rosenberg, 2000). When the temperature of the emulsions drying is high, emulsions tend to remove moisture faster, and thus, the spherical structure is formed (Nijdam & Langrish, 2006). Therefore, due to cellular morphology, it can be ensured that all moisture is removed from the nanocapsules during the drying process. If some of the moisture remains inside the nanocapsules, their structure changes from smooth to intense and rough state (Tonon, Brabet, & Hubinger, 2008). Tan, Kha, Parks, Stathopoulos, and Roach (2015) showed a similar morphology of the melan extracts in nanocapsules of maltodextrin and arabic gum coatings.

### 3.4 Oil tests

#### 3.4.1 Peroxide value

**Figure 2:**
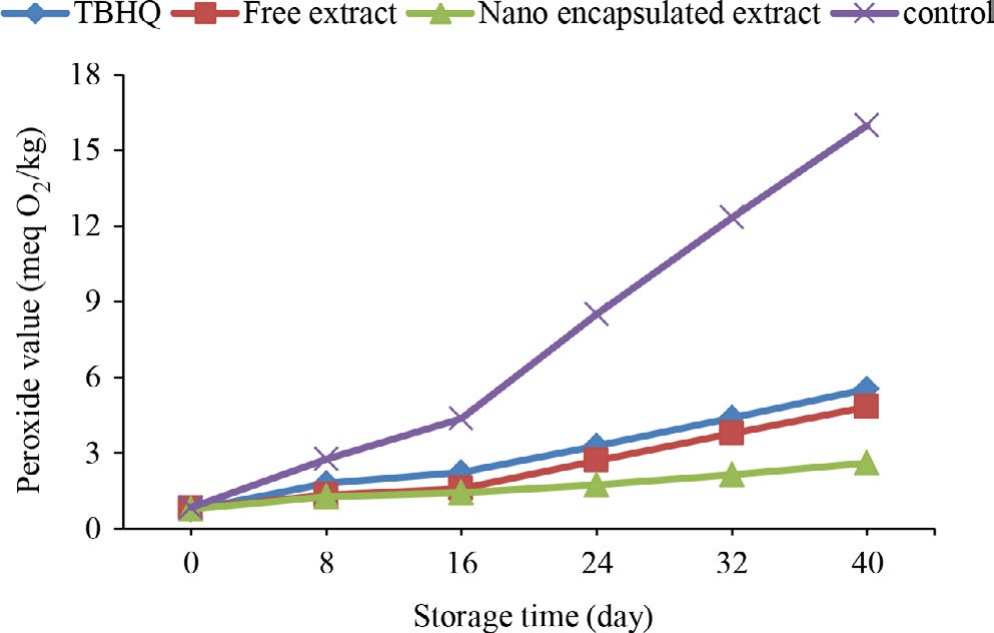
Change in peroxide value of different soybean oil samples during storage

Peroxides are the primary product of oxidation of oils and can be estimated using the peroxide index (Malheiro et al., 2013). High levels of peroxide value show less oxidative stability in oil samples (Naghshineh, Ariffin, Ghazali, Mirhosseini, & Mohammad, 2010). PV is one of the qualitative parameters and indicators related to the chemical degradation of oils and measures the concentration of peroxides and hydroxides during storage (Albu, Joyce, Paniwnyk, Lorimer, & Mason, 2004).The results of the changes in the peroxide value of different oil samples during the storage time are shown in Figure 2. It can be seen that in all investigated samples, during storage time, the peroxide value has increased in all samples and the difference of this parameter is significant at different storage times. The control sample showed the highest amount of PV. Taghvaei et al. (2014) showed that the soybean oil sample (without antioxidant) had the highest peroxide value during the storage time, and the peroxide value of these increased during storage. This increase is initially slowed and then greatly increased and is agreed with the results of this study. In the samples containing TBHQ, the pure extract, and encapsulated extract, the samples were not significantly different until the 16th day of storage, and then, there was a significant difference. The lowest amount of peroxide value was observed in samples containing encapsulated extract, pure extract, and TBHQ, respectively. Antioxidants increase the activating energy by increasing the total energy of activation and thus reduce the rate of oxidation reactions (Frankel, 1996). These results are agreed with the results of Farahmandfar, Naeli, Naderi, and Asnaashari (2019), Farahmandfar, Asnaashari, Pourshayegan, Maghsoudi, and Moniri (2018), and Sayyad and Farahmandfar (2017).

These results are consistent with the results of Yang et al. (2016). They also examined the effect of rosemary extract on soybean oil, flaxseed oil, and rice bran oil, which indicated that the control samples had higher peroxide value than the extracts and samples containing synthetic antioxidants. Bagheri, Izadi Amoli, Tabari Shahndasht, and Shahosseini (2015) determine the antioxidant effects of free and encapsulated extract of fennel in preventing oxidation of Kilka fish oil. The extract prevents the oxidation of fatty acids due to phenolic compounds, which has an antioxidant effect on the encapsulated extract. These results are agreed with the results of the present study. This indicates the ability of the encapsulation to improve the antioxidant activity of the extract and increase the availability of the extract, which has been shown in various studies (Bagheri et al., 2015).

#### 3.4.2 Measuring the Thiobarbituric Acid Value

**Figure 3:**
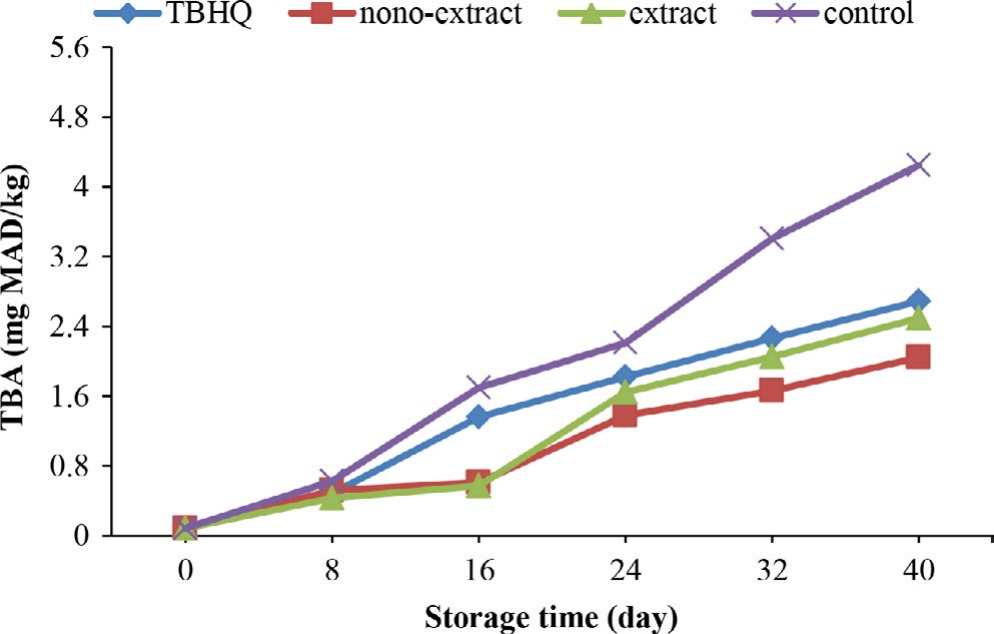
Change in thiobarbituric acid value of different soybean oil samples during storage

The peroxides produced from the first stage of oxidation are highly unstable compounds and rapidly decompose under the influence of heat and produce secondary oxidation compounds (Shahidi & Zhong, 2010). The change in thiobarbituric acid value of different oil samples during the storage time is shown in Figure 3. It can be seen that the trend of changes in all samples is increasable, and there is a significant difference between different storage times. All samples were not significantly different on day 0. There was no significant difference between the 24th and the 40th days in the storage of samples containing pure extract and TBHQ. The samples containing the encapsulated extracts had the least amount of TBA value and had a significant statistical difference with other samples. Fang and Bhandari (2010) stated that the use of encapsulation process has led to an increase in the application of encapsulated extracts, which is consistent with the results of this study. Gortzi, Lalas, Tsaknis, and Chinou (2006) showed that antioxidant activity of encapsulated extract is more than the free extracts in food systems.

## 4 CONCLUSION

Today, a wide range of antioxidants are used for various purposes in the preparation of various foods. The importance of these antioxidants is so much that without using them, the production and consumption of many items and foods are almost impossible. Most preservative compounds that are nowadays added to food as a food additive are not natural and are chemically produced or synthesized. The results of this study showed that the extract of the hyssopus extract containing phenolic antioxidant compounds, and the encapsulation process results in the preservation of the hyssopus extract during storage in the oil. Encapsulation is an effective way to increase the antioxidant activity of the extract, and if used, it can increase the shelf life of edible oils with natural antioxidants.

## CONFLICT OF INTEREST

The authors declare that they do not have any conflict of interest.

*Informed Consent*: Written informed consent was obtained from all participants.

## ETHICAL STATEMENT

Human and animal testing is unnecessary in this study.
